# Editorial: Chilling tolerance and regulation of horticultural crops: physiological, molecular, and genetic perspectives

**DOI:** 10.3389/fpls.2024.1549259

**Published:** 2025-01-14

**Authors:** Isabel Lara, Diane M. Beckles, Maria F. Drincovich, Shifeng Cao, Julian C. Verdonk, Reinaldo Campos-Vargas

**Affiliations:** ^1^ Departament de Química, Física i Ciències Ambientals i del Sòl, Universitat de Lleida, Lleida, Spain; ^2^ Department of Plant Sciences, University of California, Davis, Davis, CA, United States; ^3^ Centro de Estudios Fotosintéticos y Bioquímicos (CEFOBI), CONICET, Universidad Nacional de Rosario, Rosario, Argentina; ^4^ Zhejiang Wanli University, Ningbo, China; ^5^ Horticulture and Product Physiology, Plant Science Group, Wageningen University and Research, Wageningen, Netherlands; ^6^ Departamento de Producción Agrícola, Universidad de Chile, Santiago, Chile

**Keywords:** chilling response, chilling stress, chilling tolerance, horticultural crop, metabolomics, transcriptomics

## Introduction

Crops of tropical and subtropical origin are cold-sensitive at every stage of the lifecycle, including postharvest cold storage. Chilling-induced damage leads to diverse symptoms which manifest as external alterations (surface pitting, discolouration), internal disorders (browning, water soaking) and/or impaired physiological processes (ripening and growth inhibition, wilting, altered flavour, decay).

Plants have developed complex tolerance mechanisms to counteract or to minimize chilling injury (CI), including stress perception, signal transduction, transcriptional activation of stress-responsive target genes, and the synthesis of stress-related proteins and other molecules. The integration of molecular and *omics*-based approaches has provided new *loci* for marker-assisted breeding toward chilling tolerance. Thus, understanding the physiological, biochemical and molecular responses underlying these tolerance mechanisms, and the causal genes, is paramount to engineering strategies for enhanced tolerance to chilling stress.

This Research Topic was launched to gather recent investigations in this field. Fourteen papers were finally compiled, which examined a wide range of edible and non-edible plant species, including climacteric and non-climacteric fruit species, herbs, and edible corms or seedlings belonging to different botanical families. They explored general mechanisms involved in plant cold tolerance and the suitability of exogenous treatments for alleviating CI-related disorders.

## Mechanisms involved in tolerance to cold stress

Plants employ sophisticated molecular mechanisms to perceive and respond to cold stress through cold tolerance and/or cold avoidance. However, the specific physiological and molecular mechanisms driving these protective effects remain poorly understood. Jahed et al. surveyed recent literature on these mechanisms and their potential impact on crop productivity and sustainability. One key adaptive strategy identified was the production of cryoprotectant molecules, which have antioxidant and reactive oxygen species (ROS) activity. These processes prevent protein denaturation, mitigate cell damage and minimize cell dehydration, which preserves cellular function under low temperatures.

Sun et al. explored the biological mechanisms underlying the response to cold in the seedling roots of ‘Jinyou 35’, a cold-tolerant cucumber (*Cucumis sativus* L.) cultivar. Hormonal analysis revealed high auxin accumulation and roles for jasmonic acid and strigolactone in the cold-tolerant response. Metabolite profiling identified phenolic acids as the most abundant metabolites under chilling conditions, with potential roles for triterpenes. Seedling transcript analysis pointed to *AP2/ERF* transcription factor gene induction under both suboptimal and low-temperature stress. Interestingly, the benzoxazinoid biosynthesis pathways were upregulated at the transcript and metabolite level.

In a large-scale study, Zhang et al. investigated the metabolic mechanisms underlying the cold-induced flavour loss in melon (*Cucumis melo* L.). The key finding was the reduced emission of acetate esters due to transcriptional regulation. Several transcription factors, such as *NOR*, *MYB*, and *AP2*/*ERF*, were also suppressed. These results might provide insights into the regulation of flavour-associated pathways and the physiological chilling response in melon fruit. In addition, Lv et al. investigated the role of actin depolymerizing factors (ADFs) in different melon tissues in a low temperature-tolerant cultivar (‘LT-6’). Depolymerization of actin filaments in the cytoskeleton can improve plant tolerance to cold. Nine *ADF* genes (*CmADF*s) were identified, all responsive to temperature stress. The most responsive of them, *CmADF1*, was functionally characterized using transgenic approaches – virus-induced gene silencing of *CmADF1* increased sensitivity to low temperatures in melon. Accordingly, ectopic *CmADF1* expression in *Arabidopsis* improved cold tolerance, likely through actin filament depolymerization. The expression of *CmADF*s in abscisic acid (ABA)- and salicylic acid (SA)-treated melon leaves supports a role for *CmADF*s genes in stress responses mediated by these hormones.

Lignification may occur during postharvest storage, which in loquat (*Eriobotrya japonica* (Thunb.) Lindl.), a non-climacteric fruit, results in significant quality and economic losses. Ge et al. used transcriptomic analyses and identified the senescence-specific MADS-box gene (*EjAGL15*) as a positive regulator of postharvest flesh lignification in loquats. This study thus provided new information that may help improving loquat fruit management after harvest.

Low-temperature stress in tea (*Camellia sinensis* L.) shrubs causes growth inhibition and leaf senescence. Expression analysis by Wu et al. implicated cell wall-associated receptor-like kinase CsWAK12 in the cold response pathway. Ectopic expression of *CsWAK12* in Arabidopsis was associated with better growth but greater sensitivity to cold, and this response was linked to decreased expression of key cold-stress response genes, such as *C-repeat binding factors* (CBF) genes. CsWAK12 negatively modulates plant cold tolerance by interfering with CBF transcription while simultaneously promoting growth. Hence it may be a key sensor that redirects plant resources for growth under normal conditions, or to adaptive/tolerance responses under cold stress.

## Potential strategies for the alleviation of cold stress

There is great interest in exploring postharvest treatments that may allow the extension of storage and shelf life of commodities for higher consumption and reduced loss. Khaliq et al. applied γ-aminobutyric acid (GABA) to papaya (*Carica papaya* L.) fruit and showed enhanced oxidative stress tolerance and antioxidant defence systems in treated fruit stored under cold temperatures. GABA treatment effectively improved the chilling tolerance of papaya fruit, likely by reducing oxidative damage, which strengthened defence systems. Treated fruit were lower in lipid peroxidation, ion leakage, H_2_O_2_ content, and antioxidant enzyme activities, and had higher contents of proline, endogenous GABA, and total phenolics, which may have helped to maintain plasma membrane fluidity and integrity. Melatonin applications have shown beneficial effects against cold stress across plant species. Wang et al. applied exogenous melatonin to papaya fruit prior to cold storage and showed reduced decay compared with the untreated controls. Most ripening- and quality-related features were enhanced in the treated samples, correlating with ROS levels and enhanced activity of antioxidant enzymes. Considering increasing consumers’ concerns about the use of synthetic agrochemicals, exogenous GABA and melatonin treatments emerge as promising non-toxic alternatives to preserve papaya fruit quality during cold storage.

Dai et al. examined the potential of exogenous glycine betaine (GB) to improve tomato (*Solanum lycopersicon* L.) seedling chilling-tolerance. GB treatment activated genes related to the antioxidant system, photosynthesis, calcium signalling, energy metabolism, and cold-responsive pathways, thereby enhancing cold tolerance. GB treatment triggered higher proline content and increased activity of antioxidant enzymes while lowering the levels of malondialdehyde, an indicator of oxidative stress. Exogenous GB can act as a cryoprotectant to favour tomato seedling growth under cold stress.

Chilling injury of zucchini (*Cucurbita pepo* L.), a non-climacteric fruit species, manifests as pits, damaged areas, and fungal growth on the exocarp. Strigolactones (SL) are plant hormones that modulate plant responses to stresses, among other roles. Wang et al. showed that postharvest SL treatments improved zucchini fruit quality by maintaining membrane and oxidative status under cold treatment compared to the untreated controls. Reduced CI was associated with increased proline, arginine, ascorbic acid and ABA contents.

Paprika (*Capsicum annuum* L.), also a non-climacteric fruit species, shows CI primarily as surface pitting. Park et al. applied 20% and 30% CO_2_ shocks to mature ‘Sirocco’ paprika fruit to assess their impact on fruit quality after cold storage. Treatments preserved fruit quality during and after cold storage, with a lower incidence of surface pitting. Transcriptomic and metabolomic analyses of the fruit suggest that activation of sucrose metabolism and phosphatidic acid biosynthesis were key. These metabolites in turn induced stress signalling pathways, lipid processes and antioxidant defence mechanisms, favouring membrane stability and reducing pitting.

Basil (*Ocimum* spp.) is a high-value aromatic culinary herb with a short shelf-life, but the chilling temperatures used for postharvest preservation alter the key volatile compounds critical for desirable aroma and flavour. Rodeo et al. investigated the response of two chilling-sensitive basil genotypes (*O. basilicum* L., cv. ‘Genovese’ and *O. citriodorum* Vis., cv. ‘Lemon’) to low temperatures and atmosphere modification. Basil leaves suffered severe CI and greater loss of aroma volatiles at 5°C compared to 10°C and 15°C, which was attenuated in ‘Genovese’ but not in ‘Lemon’ samples stored under 5% CO_2_. The differentially expressed volatiles might be potential biochemical markers of chilling stress in these genotypes.

Two of the studies compiled in this Research Topic explored possible strategies to maintain the postharvest quality of Chinese water chestnuts (*Eleocharis dulcis* (Burm.f.) Trin.). Peeling the corm is necessary for consumption but leads to discoloration and quality loss. Chen et al. applied eugenol to fresh-peeled Chinese water chestnut corms, which delayed surface discolouration during storage. Eugenol enhanced antioxidant and ROS-scavenging capacity by inhibiting phenolic metabolism. In turn, Lu et al. investigated the effects of methyl jasmonate (MeJa) dips on the quality of fresh-cut Chinese water chestnuts. MeJa delayed yellowing and quality loss. Similarly to applied eugenol, MeJA treatment enhanced antioxidant capacity, inhibited ROS generation and reduced the accumulation of flavonoids, thus delaying surface discoloration.

## Conclusions

This Research Topic highlighted the conserved pathways underlying CI responses across diverse species and tissues, and illustrated the suitability of some hormonal and physical treatments to mitigate CI damage; these lines of research are necessary to support robust breeding programs and to improve postharvest management to reduce commercial losses due to CI.

